# Comparative legal perspectives on voluntary restraints: Analyzing the adaptation of preventive conditions on property rights

**DOI:** 10.1016/j.heliyon.2024.e30509

**Published:** 2024-05-03

**Authors:** Enas Mohammed AlQodsi, Iyad Mohammad Jadalhaq, Mohammed El Hadi El Maknouzi

**Affiliations:** a Department of Private Law, College of Law, United Arab Emirates University, United Arab Emirates; b Department of Private Law, College of Law, University of Sharjah, United Arab Emirates; c Mohammed V University in Rabat, Morocco

**Keywords:** Legal conditions, Legal disposition, Contract, Prevention, Invalidity

## Abstract

The difficulty of the unique nature of the conditions for preventing action, which deviates from the norm of well-known and established legal systems, has caused great differences among legal jurists. This systematic review examines the preventing condition in the French and Palestinian legal systems, analyzes the justifications presented by jurists, addressing criticisms, clarifies different directions that have emerged, and compares it with the Egyptian legislation. These countries were selected after reviewing the laws related to the phenomenon and realizing the existence of differences between them in this regard. The PRISMA protocol was followed for conducting a review of papers written by Egyptian, French or other writers, and journal articles published in reputed international journals and published in (Sciencedirect, Google Scholar, Researchgate, JSTOR and Google Books). It was conducted during July and August 2023. Moreover, studies that addressed the legal conditions that prevent action were included. The findings reflected that from the initial 431 studies reviewed, 49 met the inclusion criteria. The legal conditions that prevent action have been considered by the relatively small number of studies included in the review. Researchers from different countries examined the phenomenon, and concluded that although jurists have presented several justifications, none has succeeded in explaining the judiciary's doctrine. Accordingly, we find upon examination some jurists expressing their confusion in justifying the passage of the condition of act prevention by invalidity, and concluding that it is an unjustified course of conduct. Therefore, this study gains its uniqueness through its determination of the proper legal adaptation of the preventing condition.

## 1 Introduction

Jurists have made varying attempts to adapt the condition preventing action and directed criticism toward it [1]. However, these endeavors have not determined the legal nature of the condition preventing action. They have been criticized for trying to link the condition preventing action to one of the well-known legal systems; this link specifically is inaccurate. In this respect, the legal nature of the preventing condition is difficult to trace back to any of these systems because it has a special “self” that distinguishes it from others, and makes it “rebel” against the well-known traditional rulings. This prompted the legislator to codify what the situation has settled in the judiciary and what jurists have supported. Significantly, the condition that prevents action has its own nature, and its independent nature finds its support in the practical necessities that necessitated its existence to achieve legitimate and serious purposes. Recognizing this fact makes it easier for us to understand the provisions of the prevented condition, some of which at least represent a departure from the general rules, but an exit in harmony with its special nature. Therefore, the “self” of the condition preventing the act of the condition is nothing but an exception from the general rules, and the possibility of adapting it with reference to a legal base is doomed to failure.

The well-known traditional systems view the right of ownership as the permissibility given to the owner of the thing to freely using, investing, or disposing of it. However, the legislator may restrict this freedom when he finds it necessary to do so. Such restrictions must be mentioned in legal texts that are definitive in their meaning, because they contradict the fundamental concept of ownership.

It seems that the existence of these legal restrictions is definite from both a legal and jurisprudential standpoint. But what sparked the dispute or disagreement are the conditions that are agreed upon that prevent the owner from acting, whether they are contained in a contract binding on both sides [2], as is the case with a sale, or they are contained in an act that finds its basis in an individual will, as is the case with a will [3].

It is worth noting that contemporary jurists and jurisprudence have shown disagreement regarding the condition preventing use, as some jurists believe that the right to use is one of the basic elements of the right to property. Indeed, this characteristic has come close to the public order because it leads to the freedom of circulation of money.

### 1.1 Problem statement

The phenomenon of act prevention constitutes a serious problem that haunts the minds of legislators as it requires the availability of laws and regulations to regulate its occurrence. Different laws and legislations deal with this phenomenon differently. Therefore, the problem of this research is represented in comparing three different laws; The French Civil Law, the Palestinian Civil Law No. 4/2012, and the Egyptian Civil Code with regard to their handling of legal conditions associated with act prevention. Therefore, this research is motivated by the argument that prevention of action has been differently viewed by different researchers and legislations. It assumes that there are similarities and differences between the said laws. The three countries were selected on the basis that they locate in different continents; Europe, Africa and Asia. Though they apparently differ in terms of dealing with the phenomenon of act prevention, the French legislation has influenced the Egyptian legislation. Besides, Egypt and Palestine share the same cultural background. Significantly, the selection process occurred after reviewing the laws related to the phenomenon and realizing the existence of differences between them in this regard.

This review argues that the existing research papers on the legal conditions that prevent action have not fully examined the phenomenon in the French, Palestinian and Egyptian contexts. It also argues that the French Civil Law, the Palestinian Civil Law No. 4/2012, and the Egyptian Civil Code have certain similarities and differences in this regard. Therefore, this study attempts to answer the following questions:

1. What are the legal conditions that prevent action as they are dealt with in The French Civil Law, the Palestinian Civil Law No. 4/2012, and the Egyptian Civil Code?
2. To what extent are the selected laws similar in dealing with the legal conditions that prevent action?
3. To what extent are the selected laws different in dealing with the legal conditions that prevent action?

This study gains its uniqueness through its determination of the proper legal adaptation of the preventing condition. Its topic is unique and significant. Specifically, it is the first study to compare between The French Civil Law, the Palestinian Civil Law No. 4/2012, and the Egyptian Civil Code with regard to the legal conditions that prevent action. Therefore, it significantly highlights the reality of preventing condition adaptations and helps in shaping clear ideas about the phenomenon. Such ideas can be helpful for conducting future research with different methods and samples. It is limited to investigating the proper legal conditions that prevent action. It analyzes texts related to the phenomenon under investigation. The texts are selected from The French Civil Law, the Palestinian Civil Law No. 4/2012, and the Egyptian Civil Code. In this respect, the same phenomenon would be investigated in future research by selecting other laws dealing with the legal conditions that prevent action.

The main reason for carrying out this research is represented in determining the proper legal adaptation of the preventing condition. It is motivated by the argument that there are various adaptations of the preventing condition, some of which are illegal, and this requires conducting a systematic review of the existing knowledge for the sake of determining the legal adaptations followed to prevent action.

## 2 Methods

The inductive-deductive method is applied in this study based on a brief narration of legal texts and jurisprudential opinions in order to determine what they indicate and what they are trying to accomplish. The French Civil Law, the Palestinian Civil Law No. 4/2012, and the Egyptian Civil Code will all be compared, along with their similarities and differences, in this section. The selection of these three laws is motivated by the assumption that all of them have paid attention to the idea of legal conditions that prevent action.

Furthermore, the PRISMA protocol was followed for conducting a review of papers written by Egyptian, French or other writers, and journal articles published in reputed international journals and published in (Sciencedirect, Google Scholar, Researchgate, JSTOR and Google Books). It was conducted during July and August 2023. Moreover, studies that addressed the legal conditions that prevent action were included. The search strategy implies searching by title, reading the content or abstract, deciding whether the work is relevant to the research topic, then the final decision whether to include or exclude it is taken. The method used in selecting the source depends on whether the book or article is reviewed by other scholars. Besides, the textual method is used to prepare data for presentation. The focus was made upon comparing the legal conditions that prevent action. The initial review included 431 studies, yet only 49 met the inclusion criterion.

### 2.1 Research significance

This study gains its uniqueness through its determination of the proper legal adaptation of the preventing condition. Its topic is unique and significant. Specifically, it is the first study to compare between The French Civil Law, the Palestinian Civil Law No. 4/2012, and the Egyptian Civil Code with regard to the legal conditions that prevent action. Therefore, it significantly highlights the reality of preventing condition adaptations and helps in shaping clear ideas about the phenomenon. Such ideas can be helpful for conducting future research with different methods and samples. It is limited to investigating the proper legal conditions that prevent action. It analyzes texts related to the phenomenon under investigation. The texts are selected from The French Civil Law, the Palestinian Civil Law No. 4/2012, and the Egyptian Civil Code. In this respect, the same phenomenon would be investigated in future research by selecting other laws dealing with the legal conditions that prevent action.

### 2.2 Limitations

This research is limited to analyzing the adaptation of preventive conditions on property rights. It examines this phenomenon with reference to French Civil Law, the Palestinian Civil Law No. 4/2012, and the Egyptian Civil Code. Therefore, the future research should examine the same phenomenon as it is dealt with by laws other than the selected ones.

2. The condition preventing the act creates a state of incompetence on the part of the person stipulated.

A view in French jurisprudence holds that the condition of prevention of an act includes a reduction in the capacity of the owner, who is prohibited from acting, with regard to the behaviors that he is not permitted to perform under the same prevention condition [4].

However, this idea was devastated by a barrage of criticism, and it is no longer held. These criticisms claim that the provisions relating to capacity are considered related to public order. Besides, it is not permissible to agree to their limitation because the legislator regulates them in accordance with what is necessary for the public interest, which does not justify considering it as well. The eligibility requirements are governed by the public interest, which is guided by the legislature; however, its availability or lack thereof is not subject to special agreements [[5], [6], [7], [8]]. Apart from this, some researchers have dealt with the concept of prevention of action within the context of labour as labour discipline is included exactly as an institute that ensures this. It combines various functions - from prevention, prophylaxis and prevention of labour violations to disciplinary liability for violations [9]. Among the legal conditions highlighted by researchers is asset seizure which is associated with entrusting assets to the tribunal, in order to prevent their owner from carrying out any legal or physical act that would subtract the assets themselves [10]. Significantly, the modern forms of digitalization of labor and the increasingly aggressive use of artificial intelligence in employment relations poses in front of both legislation and doctrine qualitatively different challenges [11,12]. Furthermore, the idea of preventing action is also discussed in the context of internal audit as when external control institutions detect financial fraud and misuses, then internal audit becomes a tool for these violations, and this is a prerequisite for distorting the preventive function of internal audit [13,14].

Significantly, the legislator has stipulated the principle of the inadmissibility of relinquishing capacity or amending its provisions by virtue of a special agreement. In this context, the text of Article 48 of an Egyptian civil stipulates: “No one may waive his eligibility, nor amend its provisions. “Furthermore, Article 109 of the same law affirms this principle by saying, “Every person is qualified to contract unless his capacity is taken away or his capacity is limited by law.“It is matched in the French Civil Code by the text of Article 1123, which states: “Anyone can contract, if they are declared unable to do so by law.”

Similarly, the text of Article (57) of the Palestinian Civil Code states that “1: No one shall waive his capacity or amend its provisions, 2: Personal freedom may not be a subject of dealing.”

This view is also flawed, as it links the nature of the condition preventing the action to the capacity to perform, stating that the prevention of acting limits or detracts from the eligibility of the person prevented from acting. This is an inaccurate link, because eligibility for performance is an ability based on awareness and discrimination, while the condition of prevention from acting is based on certain considerations that have no relation from near or far with awareness and discrimination. It is recognized—without dispute—that the person who is prevented from acting is a person of full capacity in accordance with the provisions of the law [15]. This is also applicable even if we accept, for the sake of argument, that the person who is prevented from acting is incompetent or deficient in relation with the actions he is prevented from doing, he can—in accordance with the legal rules—proceed to act through his representatives or those who legally represent him, such as a guardian or trustee, and their act is considered correct, which does not apply, of course, to the prevented condition, where the person who is prohibited from acting directly cannot act on his behalf [2,4,6].

This view is criticized for considering that a person is incompetent or lacking capacity in relation to all actions, except for what is excluded by a special text [16]. Besides, if those who hold this view consider the person to be incompetent, then his actions must be voidable [2]. This is known as relative invalidity. Significantly, in case of determining the invalidity resulting from violating this condition, we will then find that the nature of this invalidity is of a special kind, as it is neither absolute invalidity nor relative invalidity. In this regard, Dr. Al-Sadda states that “The invalidity that results from lack of capacity according to the rules does not fully agree in its provisions with the intended purpose of the prevented condition. If it is absolute invalidity, it exceeds this purpose, even if it was the relative invalidity, as it falls short of achieving this goal” [4].

Another view provided by the French jurisprudence [17]argued that the condition preventing the act creates a negative obligation on the one who is stipulated to refrain from a certain action. Thus, the person is to act of the thing subject to the prevention for the duration of the period that it is prevented [18]. If the stipulated person violates the obligation imposed upon him and acts of the money subject to the prevention, the penalty is the removal of what occurred in violation, that is, this view entails the invalidity of the action. This opinion was supported by some Egyptian jurisprudence, and it significantly finds an echo in French legalization in the application of Article (1143), which states that “the creditor has the right, with an obligation to refrain, to request the removal of what has occurred”in violation of the pledge [2]. This article corresponds to Article 312 of the Egyptian Civil Code, which states: “The debtor has the right to hold against the assignee the defenses that he had to hold against the assignor at the time of the assignment's entry into force against him, and he may also hold to the defenses derived from the assignment contract.” Similarly, in the Palestinian Civil Code, Article 235 stipulates, “If the debtor commits to refrain from doing an act and breaches his obligation, the creditor may request removal of what occurred in violation of the obligation with compensation if it is necessary, and he may request permission from the court to carry out this removal at the expense of the debtor.”

Another view implies that the invalidity of the act is a pure application of these texts. This view has also drawn criticism because its logical foundation runs counter to the very concept of obligation, which presupposes the existence of two individuals, one of whom is a creditor and the other is a debtor [[19], [20], [21], [22]]. In this respect, an obligation or a personal right is “the bond of a legal relationship between two persons, which one of them (which is the creditor) can have and requires from the other (which is two debtors) a positive performance (which is an obligating to prevention).” [23].

However, this contradicts some cases in which the preventing condition is established for the benefit of the person acting himself (i.e., the owner), where the owner in this case is a creditor and a debtor of the obligation after the action at the same time. Thus, he is the owner who is obligated not to dispose of the money subject to the prevention, and he is at the same time the interest owner (i.e., the creditor) in this obligation.

Some have tried to rebut this objection by saying that it is permissible for the stipulator to demand the invalidity of the act contrary to the prevention because of his moral interest, and this interest itself is enough to say that he is the creditor of the obligation, and thus the creditor is not the debtor [7].

This position is rejected by arguing that the criterion of determining the creditor by the obligation differs from the criterion of determining who has the right to request revocation. In this respect, if only the moral interest justifies authorizing the stipulator the right to demand the annulment of the offending act, it is not—legally—sufficient to consider the stipulated a creditor of the obligation. And whoever has the right to request rescission is not necessarily the creditor, just as the primary interest on which the prevented condition was permitted and considered legally valid is the interest of the owner who is prevented from disposing of the property.

Even if we assume that the moral interest stipulated in the implementation of the impeding condition justifies his being considered a creditor by the obligation not to act in violation of this condition, then the commitment has two creditors: the stipulated and the stipulated upon. In this respect, the stipulated upon is a legal creditor and a debtor at the same time, as it has been mentioned.

Besides, this obligation depends, for its implementation in this case, on the pure will of the debtor, who is at the same time his creditor, which is illegal in terms of its consequences. It leads to the annulment of the act that includes the preventing condition if the prevented party violates the prevention requirement, which is a penalty that undoubtedly exceeds what the condition aims to achieve, and which is imposed by the stipulation of the condition itself. Prevention aims to preserve the ownership of the thing to whom it is disposed and not to take it back from him by rescinding the original disposal [24]. It is not permissible in this criticism to say that the penalty for breaching the obligation not to act is not necessarily annulment. The specific implementation, which consists in the invalidity of the violating act, may be in application of Article 212 of the Egyptian civil code corresponding to Article 1143 of the French civil code and Article 235 Palestinian civil code, which allows the creditor to request removal that carries the sense of nullity [2]. This statement has been criticized because the aforementioned article refers only to physical removal by restoring the situation to what it was before violating the obligation to prevention, such as demolishing a building that exceeded the specified height or shutting down a shop. The moral of this is that the legislator gives the creditor the right to “request permission from the judiciary to carry out this removal at the expense of the debtor.”

On the other hand, it has also been taken on this view that, according to the general rules, invalidity is the legal penalty resulting from the failure of one of the pillars of legal action or from the lack of the necessary conditions [25]in these pillars, and not the penalty for breaching a previous obligation resulting from a legal act that completed those pillars that these conditions are met. Accordingly, the statement that the act that took place in violation of the preventing condition is invalid due to the mere fact of this breach is a statement that has no basis in the law [26,27].

Numerous jurists have adapted the condition that prevents act as a burden or assignment in kind to repay a specific amount of money, making it burdened for the benefit of another person who is the owner of the interest protected by this condition, which makes it inalienable [28,29]. This view is close to the previous one in that each of them sees the prevention condition as a restriction or absurdity, but the previous view (which sees the condition of prevention as nothing more than an obligation to refrain) sees the prevention as a restriction on the shoulders of the stipulated upon [30], thus constituting an obligation. Contrastingly, the present view sees that the restriction or the burden burdens only the money itself and not the condition on it, and therefore it is a burden or a restriction in kind.

Some jurists have criticized this condition [18]. Their claim is based, on the one hand, on the idea that in rem adaptation is ambiguous, and on the other hand because its results do not respond to the intended purpose of the precluded preventing condition.

In this respect, the idea of in rem commissioning is vague and does not enable us to reach specific results. This is because jurisprudence gives it one of two meanings:

The condition of act of prevention of the property entails a new right in rem [5,19], which constitutes a criticism, implying that rights in rem have been exclusively mentioned in the law, and therefore only the legislator has the right to create new rights in rem [31]. The establishment of these rights may not be the subject of special agreements [19,32]. In this respect, when the condition of act of prevention is established for the benefit of the owner who is prevented from acting, this does not mean that the owner—in this case—has right in rem over his property [33], which is not legally proper. If this is the case, then for the sake of argument, would he not have the right to free his property from what burdens him by giving it up?Therefore, this adaptation ends with the authority for the action being distributed between the owner, who is prevented from acting, and whoever decides the condition for his benefit if he is not the owner, which is an unacceptable result, as it is not imagined that ownership of a certain thing is established for a certain person and for another having the authority to act over it, even partially. In this respect, acting is the essence of ownership, and if it is permissible for the owner to be temporarily deprived of that power, it is not conceivable that it be granted to someone else [5].

The above question can be answered by stating that the idea of burden or assignment in kind [34] is not decisive in its entirety, but that to some extent it can also be directed to the idea of voluntary restriction, proposed by critics, of the idea of in rem assignment. The most important thing taken from the proponents of the idea of voluntary restriction is that they viewed it (the idea of in-kind burden) as a right in kind, and then proceeded to direct criticism at it on this basis. In fact, the idea of a burden in kind is completely different from a right in kind. Therefore, the assignment in kind is—in fact—a burden that looks upon the money itself and not its owner, and that is why it is described as in rem, due to its link to the kind itself, not the person.

In light of this concept of the in-kind burden, the criticism is unfounded of the idea that the in-kind burden is based on the fact that the in-kind assignment confers benefits in the distribution of the power of act between the owner who is prevented from acting and whoever the condition is decided in his favor, if he is not the owner. It is not a right until it leads to the deduction of part of the owner's powers and granting it to another person. Rather, the owner's powers remain as they are without deduction [35], but with the imposition of a mandate that limits the extent of these powers. Hence, the assignment or the in-kind burden does not entitle a person other than the owner to any amount of powers until it is stated that it leads to a deduction of some of the powers of the owner, as the in-kind burden limits or restricts the powers of the owner and does not grant any of them to others.

Furthermore, the question of how a person can determine a right or a physical burden on his property can simply be answered by stating that the burden in kind is not a right until it is said that a person has a right over his property [36], and therefore this question is incorrect. But if we consider the assignment in kind as a constraint concerning money, then the same observation corroborates the statement that the preventing condition is a voluntary restriction. In this respect, the restriction imposes on the owner an authority sometimes in his favor, if the prevention is a condition in favor of the owner who is prevented from acting. This indicates limiting the owner's authority to act in order to achieve his legitimate interest, as if he were a minor and it was stipulated that he not act in order to protect him from his lack of experience and incomplete maturity. Conversely, if we take the second meaning, which is that the idea of assignment in kind means that it is the taking out of the money that is prevented being acted upon from the dealing department [37], this money becomes unfit to be a subject of financial rights, i.e., as some say, money is printed in kind, which is that it is inalienable [3,23].

However, the view has been advanced that it does not meet the general legal rules that prevent the money from being taken out of the dealing circle except by law for considerations related to the public interest, and it is not permissible to leave to the intent of individuals the power to print any money of this nature to take it out of the dealing circle. In this regard, the jurist Teissier states: “Removing money from the circle of dealing with the intent of individuals is legally impossible” [38].

In the same context, Lepelte states that “The condition of inoperability is a legally impossible condition, because it prevents money from being negotiable” [20]. Another significant criticisms against this view implies that the condition preventing the act does not remove the funds subject to the prevention from the circle of dealing. Rather, these funds remain subject to a private property right [39], meaning that they remain a valid subject of the right of ownership despite the existence of this condition and despite the inability to act of it. Likewise, its owner, although he does not have the right to act of it, can entail on this money some rights that do not contradict the condition preventing the act of this money, and this makes it unlike money outside the circle of dealing, as it is not suitable as a place for any right and it is not permissible that it result in consequences to other's rights. In this regard, Dr. Hassan Kira states that“It is established that the law has not removed the things that are forbidden to be acted of from the circle of dealing, with evidence that it still considers them, despite the prevention of acting of them [40], a subject of financial rights. And if it has given the will powers to decide to prevent the act of these things, for a certain period of time, this authority is limited to this purpose and cannot be exceeded in any case by depriving these things of their nature, by denying their validity to be the subject of financial rights” [30].

The researcher believes that the condition of inoperability is a legally impossible condition, because it prevents money from being negotiable, and this obviously indicates points of similarity between the current study and the previous studies [20,36]. Significantly, the ownership may also be transferred by a voluntary legal act, as is the case when substituting under the same condition, or with the permission of the judge in case of necessity or if the interest so requires, as we shall see.

Likewise, those who say that the money subject to the condition of being prevented of act is outside the circle of dealing accept that the penalty for violating the condition preventing the act is the absolute nullity of these disobedient behaviors. This is because every act of money outside the circle of dealing is rejected as absolutely null and void. This logical sequence is not consistent with what has been determined by French and Egyptian jurisprudence, as we will come to the conclusion that absolute invalidity is not appropriate as a penalty for violating the condition preventing action [3,41].

To sum up, adapting the condition that prevents [42]act as a burden or an obligation in kind to return the money to which this condition applied is a view that many jurists have advanced, and it still has its supporters and defenders. But despite the failure of definitive responses to these criticisms, the jurisprudence supporting this trend has tended to combine this idea with the idea that we will discuss in the next paragraph related to considering the condition preventing act as being an amendment to the property system.

Dr. Mansour Mustafa Mansour states that “We do not find a difference between the view we prefer [assignment in kind] and what his critics conclude that the condition of prevention from act leads to an amendment in the provisions of ownership or to a limitation on the owner's powers” [7]. The first to advance this adaptation are two French jurisprudents (Ribbipropoulanje) and some Arab jurists followed them in this direction. Dr. Al-Sadda states that “The correct thing is that the condition of prevention from action includes a restriction on the powers of the owner, as it includes an amendment in the ordinary system of ownership according to the intent of individuals” [19]. From the point of view of the proponents of this view—that it is merely an exception or a departure from what the property originally confers on the authority to act of the money subject to the right—it is a voluntary modification of the property system for a temporary period in order to achieve a legitimate interest. The law is the entity that has given the intent this power to modify the property system, so this modification must be within the limits that are consistent with the intended interest, and therefore the preventing condition in its provisions is subject to the requirements of this interest, so that these provisions differ, according to this interest [43], from one case to another [5]. This study agrees with these points that indicate the scholars' view of preventing condition as a voluntary modification of the ordinary property system [5,7,19,41].

So long as this condition is viewed as nothing but an amendment to the property system constituting an exception to the original rule that allows the owner to act of his money, it remains dependent on its provisions and its effects on the purpose of its requirement, that is, the end for which this condition was permitted to protect the aimed interests. Moreover, the penalty for violating this condition should not be subject—based on this conditioning—to fixed rules that do not change. Rather, it must differ from one case to another, depending on the purpose of this condition, as the law did not authorize the will such an exceptional authority to modify the property system and deprive the owner of his authority to act for a temporary and reasonable period, except as a guarantee to achieve special interests in modifying the property system, so the penalty for violating this amendment or deprivation—voluntary—by the occurrence of the act despite the prevention of it—must be sought within the limits of this purpose alone and to the extent that guarantees its achievement.

In this respect, Boudry claims that the non-effectiveness of the act against the one who obtained the prevention in his favor, rather than its invalidity, is the consequence of violating the condition preventing the act [44].

Obviously enough, this view is not completely perfect. Rather, it has been said that adapting the condition that prevents them from acting in this way means arranging rights for people who no longer have rights. When it is said that the action is not effective in the face of the first acting person, here it means that the latter is still the owner of the money, or that he has a right in kind over this money at a time when the idea of the existence of such a right was excluded when responding to those who say that there is a “commitment in kind.” In this respect, it is truly noted that “assuming that the contracting party who stipulated not to act is no longer the owner of the money, and he does not have any other right in kind, how is the action considered ineffective against him?” [34].

Therefore, it is rightly argued that this view is not proper, because the non-performance of the exchange is not limited to the conditional or the first acting person only [45], but extends to include whoever the stakeholder is, whether the acting person himself or someone else. In this context, to say that the first acting person, against whom the action is not effective, may actually have rights over this money, as if the intent of the actor by imposing this condition is to ensure the payment of the deferred price and to prevent this money from coming out of the act of the actor to him before paying its price. The enforcement of the action against him is considered feasible, as he reserves his right to reimburse the price when the person acted to him acts in contravention of the provisions of this condition.

## 3 Discussion

### 3.1 Discussion of the findings related to the first question “what are the legal conditions that prevent action as they are dealt with in The French Civil Law, the Palestinian Civil Law No. 4/2012, and the Egyptian Civil Code?”

According to French jurisprudence, the condition of prevention of an act is associated with a reduction in the capacity of the owner, who is prevented from acting. In this respect, asset seizure is one of the legal conditions highlighted by researchers and it associated with entrusting assets to the tribunal, in order to prevent their owner from carrying out any legal or physical act that would subtract the assets themselves. Another condition is associated with internal audit as when external control institutions detect financial fraud and misuses, then internal audit becomes a tool for these violations, and this is a prerequisite for distorting the preventive function of internal audit. These conditions are also dealt with by the Egyptian and Palestinian laws that regard the conditions preventing an action as indications of a reduction in the capacity of the owner.

### 3.2 Discussion of the findings related to the second question “to what extent are the selected laws similar in dealing with the legal conditions that prevent action?”

All the three laws significantly agree with regard to linking the nature of the condition preventing the action to the capacity to perform, stating that the prevention of acting limits or detracts from the eligibility of the person prevented from acting. In this respect, the French law implies that the condition preventing the act causes a negative obligation on the one who is stipulated to refrain from a certain action. Interestingly, some Egyptian jurisprudence hold the same opinion as it agrees with the French legalization in the application of Article (1143).

The same thing is applicable to the Palestinian Civil Code, which is evident in Article 235 that stipulates, “If the debtor commits to refrain from doing an act and breaches his obligation, the creditor may request removal of what occurred in violation of the obligation with compensation if it is necessary, and he may request permission from the court to carry out this removal at the expense of the debtor.”

Another point of similarity is associated with the specific implementation of articles related to this issue. This consists in the invalidity of the violating act, and it may be in application of Article 212 of the Egyptian civil code corresponding to Article 1143 of the French civil code and Article 235 of the Palestinian civil code, which allows the creditor to request removal that carries the sense of nullity.

### 3.3 Discussion of the findings related to the third question “to what extent are the selected laws different in dealing with the legal conditions that prevent action?”

The major differences between the three selected laws, with regard to the conditions preventing an action, were reflected in the organization of the laws on this subject. Despite the fact that all the three laws provide texts that aim to regulate the conditions preventing an action, they differed with regard to the principle of the condition preventing action. These different positions are supported by different arguments as well, which prompts the researcher to study and investigate. Each law presents different conditions associated with different issues of preventing an action.

## 4 Our preferred view

The foregoing analysis obviously reflects the correctness of the latter view, which considers the condition preventing the act a departure from the general provisions of ownership in order to protect the legitimate interests of the people. This is because it constitutes a restriction on the right of ownership and detracts from the powers of the owner. Besides, the modification in the property system is nothing but an exception to the original rule that allows the owner to act of his money.

In fact, adapting the condition that prevents us from acting in this way, as we think, is not an adaptation in the exact sense, because adapting means rooting the idea by returning it to one of the general rules in the law. Therefore, the assumption that this condition is an exception to all these rules indicates that the condition preventing action cannot be attributed to any of these rules, and therefore it cannot be adapted. This is the reality of the case, as this condition is nothing but an exception to these rules, and we have seen how all the attempts that tried to bring it back to the general rules of law failed because they tried to insert this condition into these rules at a time when this condition had nothing to do with them. The legal text in general is not a basis for any legal idea [46], but rather a source for it [47]as well as its legislative support. As for its legal basis, it is something completely different. Here, it means “the rooting for this idea, and an attempt to return it to one of the well-known legal systems [48], or to create an appropriate system, to which it can be attributed, if it is difficult to return it to any of these systems” [[49], [50], [51]].

Adapting the condition that prevents acting as a modification or exception to the general rules of ownership and as having its own nature and independent entity finds support in the practical necessities that imposed its existence with the aim of achieving legitimate purposes. It is very useful in arranging the provisions of this condition, its effects, and the penalty for violating it, in order to free it from all restrictions and provisions that would restrict it were it linked to one of the legal systems indicated by the general rules.

Thus, the condition that prevents action is what we see as a voluntary in-kind burden that looks upon the money subject to the prevention in order to achieve the legitimate interest intended behind the prevention.

## 5 Conclusion

Economic life requires freedom of circulation of money. This view was present in the judiciary, which stressed prohibiting the condition preventing action and considering it a fundamentally invalid condition. But the practical reality and its implications and the necessity of distinguishing between the prohibitive condition contained in negotiations and the prohibitive condition contained in donations began to impose itself when many cases and applications emerged, implying that the prohibition of disposal is based on legitimate justifications and motives, which does not entail with the condition any loss of the right of ownership and the freedom of circulation of funds.

These differences were reflected in the organization of the laws on this subject, as they differed with regard to the principle of the condition preventing action. These different positions are supported by different arguments as well, which prompts the researcher to study and investigate.

Significantly, jurists hold different views concerning the way that they adapt and criticize the condition preventing action. They continue to make efforts to ascertain the legal status of the condition preventing action. These attempts have drawn criticism because they attempted to relate them to well-known legal systems, and this particular link is inaccurate. Because the prevention condition has a unique “self” that sets it apart from others and causes it to “rebel” against the well-established conventional rules, it is challenging to trace the legal nature of the prevention condition back to any of these systems. This prompted the legislator to codify what the judiciary had decided and what the legal community had agreed upon.

## Data availability statement

Data sharing is not applicable to this article as no new data were created or analyzed in this study.

## Ethical approval

This article does not contain any studies with human participants performed by any of the authors. Instead, this work is based exclusively on desk-based research, relying on commentary from legal texts and scholarly documents that are freely available in the public domain. As such, it presupposes no activity for which an ethical approval would be needed at the Authors’ academic institutions.

## Financial support

This study is self-financed.

## Informed consent

Informed consent was not required for this study because this article does not contain any studies with human participants performed by any of the authors.

## CRediT authorship contribution statement

**Enas Mohammed AlQodsi:** Writing – review & editing, Writing – original draft, Visualization, Validation, Supervision, Software, Resources, Project administration, Methodology, Investigation, Formal analysis, Data curation, Conceptualization. **Iyad Mohammad Jadalhaq:** Writing – review & editing, Methodology, Data curation, Conceptualization. **Mohammed El Hadi El Maknouzi:** Writing – review & editing, Methodology, Formal analysis.

## Declaration of competing interest

The authors declare that they have no known competing financial interests or personal relationships that could have appeared to influence the work reported in this paper.

## Originality

This manuscript has not been published previously, and that it is not under consideration for publication elsewhere, in full or in part.
